# A low-cost platform for automated cervical cytology: addressing health and socioeconomic challenges in low-resource settings

**DOI:** 10.3389/fmedt.2025.1531817

**Published:** 2025-03-31

**Authors:** José Ocampo-López-Escalera, Héctor Ochoa-Díaz-López, Xariss M. Sánchez-Chino, César A. Irecta-Nájera, Saúl D. Tobar-Alas, Martha Rosete-Aguilar

**Affiliations:** ^1^ Departamento de Salud, El Colegio de la Frontera Sur, San Cristóbal de las Casas, Chiapas, México; ^2^ SECIHTI - Departamento de Salud, El Colegio de la Frontera Sur, Villahermosa, Tabasco, México; ^3^ Departamento de Salud, El Colegio de la Frontera Sur, Villahermosa, Tabasco, Mexico; ^4^ Hospital General de Zona No. 2, Instituto Mexicano del Seguro Social, Tuxtla Gutiérrez, Chiapas, México; ^5^ Instituto de Ciencias Aplicadas y Tecnología, Universidad Nacional Autónoma de México, Circuito Exterior S/N, Cd. Universitaria, México City, México

**Keywords:** cervical cancer, cervical cytology automation, digital microscopy, low-cost diagnostics, low-resource settings, point-of-care diagnostics, AI in cervical screening

## Abstract

**Introduction:**

Cervical cancer remains a significant health challenge around the globe, with particularly high prevalence in low- and middle-income countries. This disease is preventable and curable if detected in early stages, making regular screening critically important. Cervical cytology, the most widely used screening method, has proven highly effective in reducing cervical cancer incidence and mortality in high income countries. However, its effectiveness in low-resource settings has been limited, among other factors, by insufficient diagnostic infrastructure and a shortage of trained healthcare personnel.

**Methods:**

This paper introduces the development of a low-cost microscopy platform designed to address these limitations by enabling automatic reading of cervical cytology slides. The system features a robotized microscope capable of slide scanning, autofocus, and digital image capture, while supporting the integration of artificial intelligence (AI) algorithms. All at a production cost below 500 USD. A dataset of nearly 2,000 images, captured with the custom-built microscope and covering seven distinct cervical cellular types relevant in cytologic analysis, was created. This dataset was then used to fine-tune and test several pre-trained models for classifying between images containing normal and abnormal cell subtypes.

**Results:**

Most of the tested models showed good performance for properly classifying images containing abnormal and normal cervical cells, with sensitivities above 90%. Among these models, MobileNet demonstrated the highest accuracy in detecting abnormal cell types, achieving sensitivities of 98.26% and 97.95%, specificities of 88.91% and 88.72%, and F-scores of 96.42% and 96.23% on the validation and test sets, respectively.

**Conclusions:**

The results indicate that MobileNet might be a suitable model for real-world deployment on the low-cost platform, offering high precision and efficiency in classifying cervical cytology images. This system presents a first step towards a promising solution for improving cervical cancer screening in low-resource settings.

## 1 Introduction

**Figure 1:**
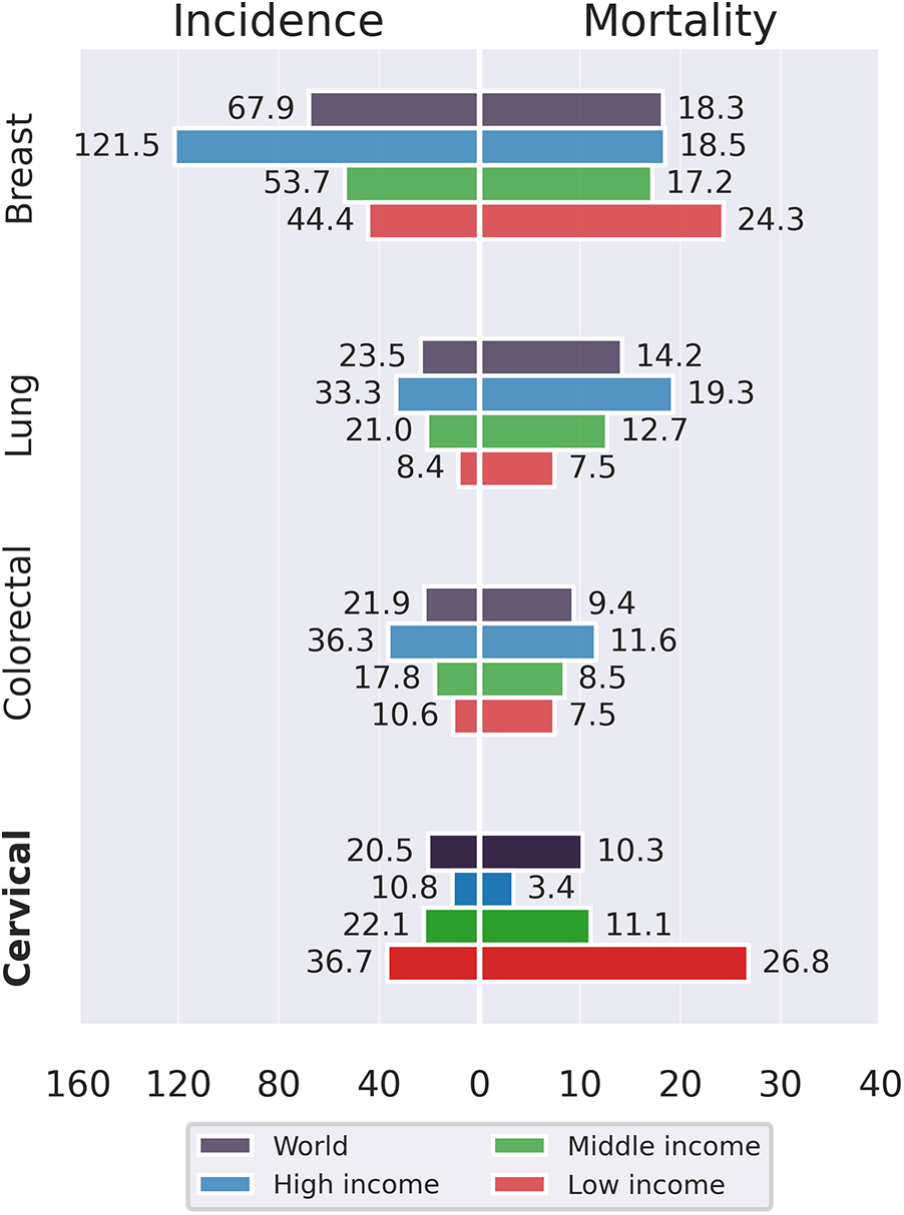
Incidence and mortality rates (ASR) of cancers in women aged 15 and above categorized by income levels across countries (1).

Cervical cancer (CC) continues to pose a substantial threat to global health due to its high morbidity and mortality burden. In 2022 alone, this disease presented around 660,000 new cases and claimed the lives of around 350,000 women globally, making it the fourth most common and third most fatal cancer among women (1). However, as illustrated in Figure 1, CC disproportionately affects women in low- and middle-income countries (LMICs), standing out as the leading cause of cancer-related deaths among women in low-income nations. This disparity can be attributed to various factors, including cultural aspects, scarce resources, inadequate infrastructure, and a shortage of skilled healthcare professionals in these areas (2).

The numbers described above contrast with the fact that CC is a preventable and treatable form of cancer if detected in early stages. This disease, primarily caused by a persistent infection of human papillomavirus (HPV), progresses slowly over a period of about 10 to 20 years, providing a window of opportunity for detection and intervention (3). In this context, the implementation of efficient screening programs is crucial to combat the high incidence and mortality rates associated with CC. Cervical cytology, commonly known as the Pap test, remains the most widely used method for cervical cancer early detection in LMICs (4). During a conventional Pap test, a healthcare provider collects cells from the cervix using a brush or spatula and deposits them onto a microscope slide. The sample is then fixed, stained, and analyzed under a microscope by specialists in a laboratory to detect abnormalities such as precancerous changes, cancerous cells, or signs of infections including HPV (5). This traditional framework for implementing cervical cytology screening, although proven to be really effective in some regions (6), is labor-intensive, time-consuming, and reliant on skilled cytologists for accurate interpretation. This challenge has led to several efforts to automate the process.

The first attempt at automating cervical cytology screening emerged nearly 70 years ago with the development of the *Cytoanalyzer*, which aimed to reduce reliance on manual screening (7). This device combined a conventional microscope with a photomultiplier to convert optical information into electrical signals, which was reportedly able to distinguish normal from abnormal cervical cells. Later, during the late 1970s and 1980s, advances in digital technology led to the emergence of several automated cytology projects (8–13). These systems shared a common approach: extracting cellular features from digital images to differentiate normal from abnormal cells. Key features analyzed included nuclear area, nuclear density, cytoplasmic area, and the nuclear-to-cytoplasmic ratio. However, none of these devices reached the market due to their lack of cost-effectiveness (14). In the late 1980s, innovations in sample preparation significantly improved specimen quality, making feature extraction easier (15, 16). However, it was not until the late 1990s that the first commercial automated screening systems received approval from the U.S. Food and Drug Administration (FDA). In practice, achieving zero false negatives and zero false positives is impossible, making the performance requirements for screening machines a long-standing point of controversy. To this day, there is no consensus on how to evaluate a screening system before approving it for routine use (14). Currently, the only two FDA-approved commercial systems—the ThinPrep Imaging System and the BD FocalPoint GS Imaging System (17)—still require human supervision. While both have demonstrated high effectiveness, their substantial purchase and operational costs—reportedly in the range of tens of thousands of U.S. dollars—make them impractical for large-scale screening programs in LMICs.

In the previous context, open-source hardware development offers an interesting alternative. Open-source hardware refers to designs made publicly available, allowing anyone to study, modify, distribute, manufacture, and sell both the design and its resulting hardware (18). This approach, combined with the rise of digital fabrication technologies—such as affordable 3D printing, CNC machines, and laser cutters—along with accessible microcontrollers like Arduino and Raspberry Pi, has significantly boosted local hardware development. Furthermore, institutional support from universities, research centers, and companies has further propelled these advancements. This movement has extended to medical device development, resulting in open-source alternatives for various types of medical equipment (19, 20). However, to our knowledge, no direct attempts have been made on open hardware for automated cervical cytology. At their core, these devices rely on automated microscopes paired with image analysis algorithms.

Commercial automated microscopy systems are expensive, limiting their adoption in low-resource settings. However, over the past decade, several open-source microscopy devices have been developed, making advanced microscopy more accessible. One of the most widely recognized examples is OpenFlexure (21), a fully automated, 3D-printed laboratory microscope featuring motorized sample positioning and focus control. Other notable open-source microscopes include FlyPi (22), UC2 (23), and µSmartScope (24). However, certain limitations hinder their applicability for Pap smear analysis in low-resource settings. The most critical limitation is their scanning surface coverage. Pap smears are typically spread over a standard microscope slide, covering an area of approximately 50 × 25 ${\mathrm{mm}}^{2}$. For instance, FlyPi lacks an automated scanning function, while OpenFlexure and µSmartScope have limited scanning ranges that fail to cover the entire required area. Although UC2 is developing a large-area scanning platform, it relies on components that may not be readily available in low-resource environments. These constraints highlight the need for further innovations in open-source microscopy to enable large-scale, automated cervical cytology screening.

In the field of image analysis, almost all early approaches to automated Pap smear interpretation followed a similar workflow. The process began with cell or nucleus segmentation, followed by manual feature design (nucleus area, cytoplasm area, nucleus perimeter, cytoplasm perimeter, nucleus-to-cytoplasm ratio, among others). The extracted hand-crafted features were then fused and used for final classification (25, 26). However, in recent years, deep learning (DL) algorithms have transformed the field, significantly improving the ability to recognize complex patterns in images (27). Unlike traditional methods, deep learning models—particularly convolutional neural networks (CNNs)—can automatically learn informative and meaningful features directly from raw image data, eliminating the need for explicit cell segmentation and manual feature engineering (28). This breakthrough has led to a surge in research on DL-based automated cervical cytology analysis, with numerous studies exploring different algorithmic approaches, imaging modalities, and sample preparation techniques (17). Notable results have been achieved with transfer learning algorithms, which leverage pre-trained architectures that are subsequently fine-tuned on custom datasets. In the context of cervical cytologic analysis, Wang et al. achieved accuracies exceeding 98% for two-class classification on a custom dataset (29), while Khamparia et al. reported accuracies above 99% for two-class classification using the Herlev dataset (30). Despite these promising results, significant challenges remain, including recognition of additional relevant classes, generalization across populations, and integration into clinical workflows. Addressing these issues will be essential for transitioning DL-based solutions from research to real-world deployment, particularly in LMICs where cost-effective automation could have the greatest impact.

To our knowledge, the only study proposing a non-commercial, fully integrated approach—combining an automated imaging system with DL algorithms for cervical cancer screening—is that of Holmström et al. (31). Holmström and colleagues used the commercial Grundium Ocus slide scanner to digitize whole Pap smear slides. They trained and tested a DL algorithm to detect slides with low-grade and high-grade lesions. The model achieved sensitivities and specificities of 84.2%, 86.0% for low-grade lesions, and 85.7%, 98.5% for high-grade lesions. These results are promising for potential implementation in low-resource settings, particularly if the approach is expanded to include classification to additional relevant cancer stages. However, large-scale adoption in low-resource settings may require more affordable slide-scanning solutions.

Finally, conventional Papanicolaou tests must be considered within the broader context of modern cervical cancer elimination strategies. HPV vaccines are expected to significantly reduce cervical cancer incidence, as they provide exceptional protection against high-risk HPV infection (32). However, even with the most effective vaccination programs, these will take decades to be fully realized (33), and in the meanwhile millions of women remain at risk. Additionally, HPV vaccines remain expensive, which can be prohibitive in low-resource settings. Concerns also exist regarding vaccine-induced strain replacement, which may affect long-term efficacy (34). At the same time, high-income countries are shifting their screening focus toward molecular HPV testing due to its high sensitivity and specificity (35). While these tests may offer advantages in early detection, they are costly and require specialized infrastructure, making their implementation challenging in rural and underserved areas. Given these challenges, conventional Pap tests remain essential for cervical cancer prevention, but innovative point-of-care (POC) diagnostic solutions are needed (36). In this context, this research paper describes the development of a low-cost platform for automated cervical cytology, using a novel approach that combines a custom made low-cost automated microscope with deep learning algorithms.

## 2 Materials and methods

### 2.1 Device overview

**Figure 2:**
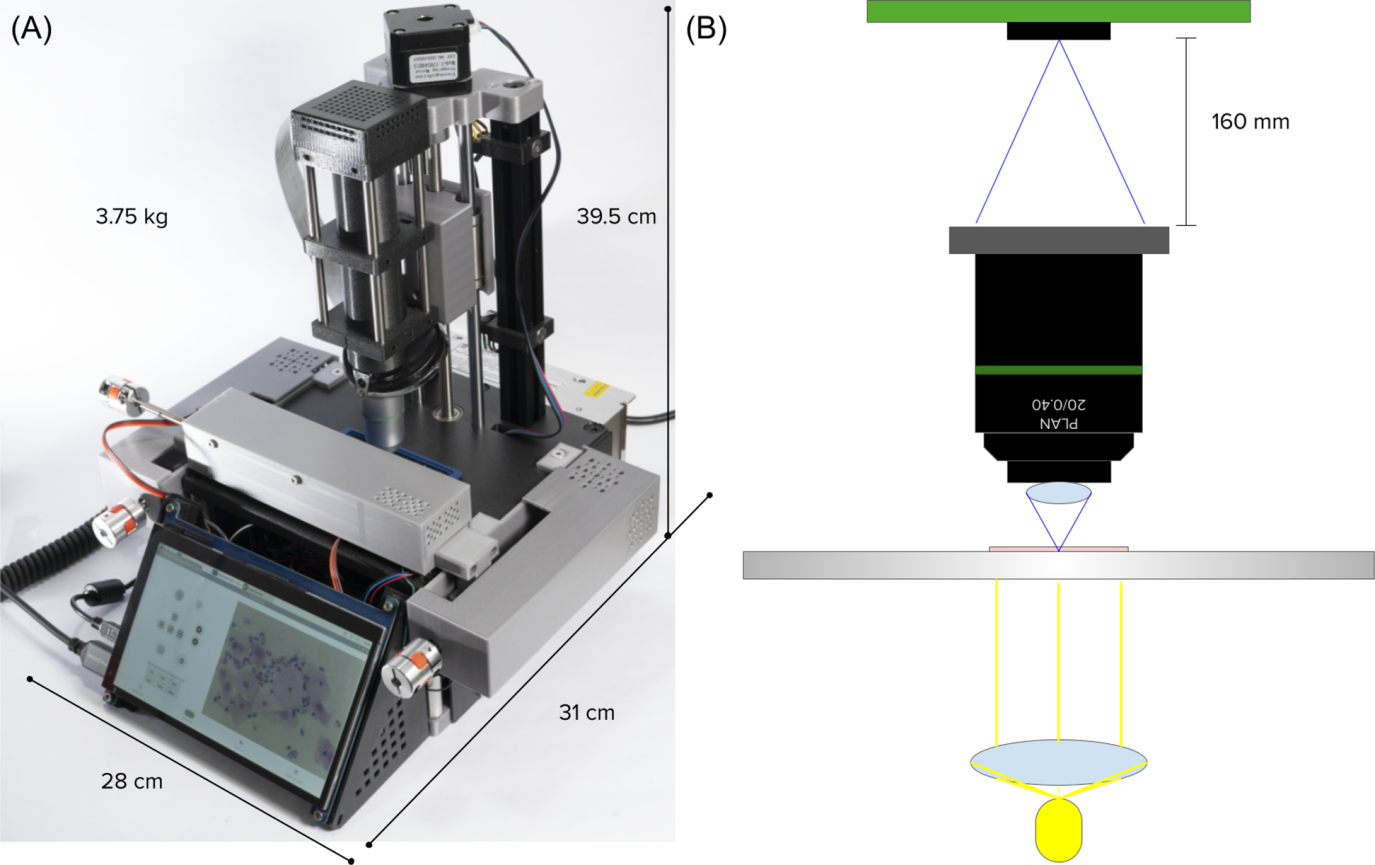
**(A)** Picture of the platform. **(B)** Scheme of the optical setup.

In this section, we describe the general characteristics of the developed microscopy platform, which is shown in Figure 2A. The design and development of the platform were executed with a focus on a couple of key aspects essential for its successful implementation in resource-constrained settings. It is important to highlight that this project was developed in Chiapas, the poorest state in Mexico (37). Visits and interviews conducted at rural health centers provided valuable insights into the challenges faced for effective cervical cancer screening in rural communities with low resources. In the first place, the platform was engineered using non-sophisticated and low-cost components that are increasingly becoming more available in LMICs (38), ensuring accessibility and affordability. On the other hand, ease of operation was an important consideration, enabling straightforward usage by health personnel with limited technical expertise. Additionally, a focus on ease of construction and repairment was paramount during the development phase, enabling reproducibility by people with little tinkering abilities as well as quick and cost-effective maintenance. The design and detailed instructions for building the platform will be made available under an Open source license here, in order for anyone to be able to replicate it.

#### 2.1.1 Optics

The optical setup, shown in Figure 2B, was designed for brightfield microscopy and high-quality digital image capture, utilizing the simplest possible configuration to ensure ease of construction and repair. In the first place, we have a LED-based illumination system which utilizes a 5W high power LED and a condenser polymethylmethacrylate (PMMA) lens with a focal length of 5 mm for collimating light. Following, we use a Newport MVC-20X finite conjugate objective with a numerical aperture of 0.4, for high-quality magnification. Cheaper generic versions were also tested and sufficient acceptable qualities were achieved. Finally, for image capture, we use a Raspberry Pi HQ camera which comprises a 12.3 megapixels Sony IMX477R sensor. All parts are attached and kept aligned by an optical cage built with 3D printed parts and 6 mm steel rods. This configuration allows us for an optical resolution of 0.63 µm.

Cytological analysis involves examining features of the cytoplasm and nucleus of cervical cells (39). The smallest structures under examination are the nuclei and the chromatin spots within them. Typically, the nuclei of intermediate cells measure around 8 µm in diameter, while those of superficial cells are approximately 4 µm (40). Chromatin spots within these nuclei can range from 1 to 2.5 µm in size. Given that our optical system has a resolution of 0.63 µm, we can confidently ensure that it meets the necessary imaging specifications for accurate cytological analysis.

#### 2.1.2 Mechanics and electronics

The core electronics of the system consist of a Raspberry Pi 4 B+ with 8 GB of RAM, which controls all other electronic components. The movement system is powered by NEMA 11 and NEMA 17 stepper motors, combined with TMC2208 drivers and leadscrew mechanisms, achieving a mechanical resolution of 0.3 µm and enabling automated scanning across a $50\times 25\times 40\;{\mathrm{mm}}^{3}$ volume along the XYZ axes. After applying backlash correction by adding extra steps when changing direction, a positional repeatability accuracy of 10–20 µm in one dimension was achieved. Other electronic components include a 5 W LED with a 1 K potentiometer for light intensity control, joysticks for intuitive XYZ movement, limit switches for edge detection, and cooling fans. A custom-designed printed circuit board (PCB) simplifies connections between these components, ensuring integration and reliable operation.

The key mechanical components include aluminum T-slot profiles, steel rods, linear bearings, and trapezoidal leadscrews, all of which ensure precise alignment and smooth linear movement along the XYZ axes. Additional structural and mechanical parts were designed and 3D printed using PLA material on a Prusa MINI+ 3D printer.

#### 2.1.3 Software

**Figure 3:**
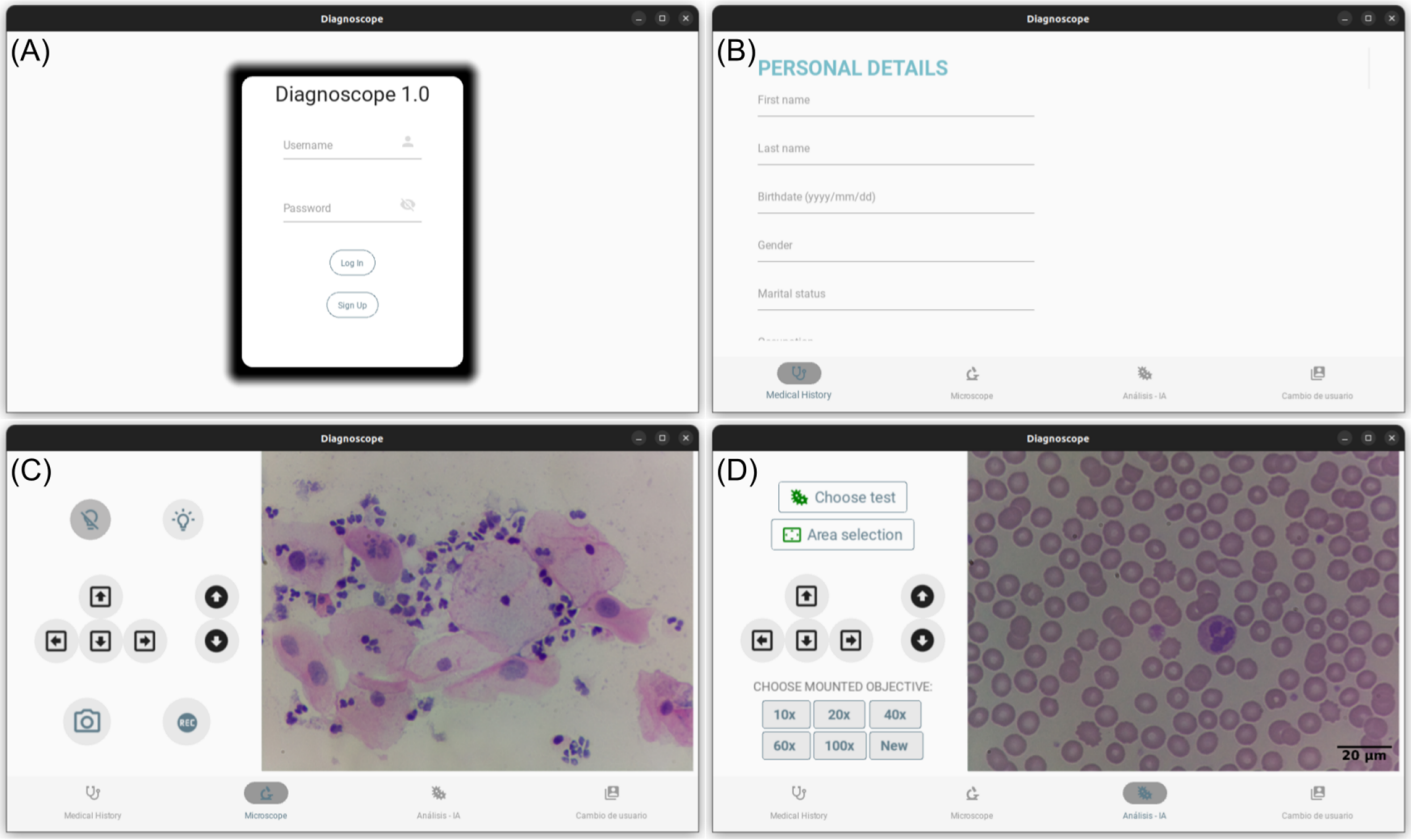
Image captures of the different sections of the software of the platform. **(A)** User login panel. **(B)** Medical record panel. **(C)** Real-time visualization panel. **(D)** Image analysis panel.

A cross-platform software for operating the device was designed with user-friendliness in mind, recognizing the lack of specialized technicians in the intended deployment environment. Both the front-end and back-end were developed using Python and will be made available under an Open Source license. The front-end, a graphical user interface (GUI), provides control for basic microscope functions, like: real-time visualization of microscopy images, autofocusing, full slide scanning capturing images of all fields of view (FoVs), and video capturing, and illumination control. Additionally, there are dedicated sections, see Figure 3, for medical history storage and for executing the image analysis module.

The software runs on a Raspberry Pi which allows the use of other resources of the operating system (OS). Particularly, the platform can be connected to the internet and upload data and images to the cloud, allowing for telemedicine practices. Additionally, the platform integrates a touch-screen which allows it to operate all functions without the need of an external computer. The software incorporates a trained CNN for automated analysis and classification of Pap smears images. A detailed description and discussion of the CNN algorithms will be provided in the following section.

### 2.2 Image analysis algorithms

CNNs stand out as a powerful approach for image analysis tasks due to their ability to automatically extract intricate features from visual data. These neural networks excel at capturing spatial hierarchies within images, making them well-suited for tasks like medical image classification where subtle details play a crucial role in accurate diagnosis (41). Consequently, in recent years, there has been an increase in the amount of researches using CNNs for medical image analysis, including automated assisted screening methods for cervical cancer (42). In the context of cervical cytology, two of the most common approaches based on CNNs are: image classification and object detection (25). Image classification models categorize an entire image into a predefined class, while object detection models search relevant objects in an image and simultaneously locate them and predict their categories. In this work we perform image classification, but not on single cell images as most works do (25), but on whole field of view (FoV) images, which may contain several cells, undertaking an approach similar to the one followed by Hussain et al. (43) and Alsalatie et al. (44).

Training a CNN which may contain millions of parameters from scratch, using only our reduced dataset can bring several issues, like overfitting and poor generalization performance. Transfer learning, a technique that attempts to transfer learned knowledge from a large public dataset to a specific task, has proved to be effective in these cases (45). Training first with a large general dataset allows to benefit from learned representations of generic features like edges, textures, shapes, etc. and further continue training making fine adjustments with a custom dataset. In this work, we tested several widely used architectures, pretrained on the ImageNet set. The selection of architectures was based on previous studies demonstrating their effectiveness, or that of related architectures within the same family, in the context of cervical cytology classification (46, 47). The tested architectures include: MobileNet, MobileNetV2, MobileNetV3Small, MobileNetV3Large, InceptionV3, DenseNet201, ResNet50, VGG16, VGG19, EfficientNetB0, and EfficientNetV2S. In order to adjust these pre-trained models to our cervical cells images classification task, we performed a fine-tuning using a custom dataset, which will be described in the following section.

### 2.3 Data acquisition

**Figure 4:**
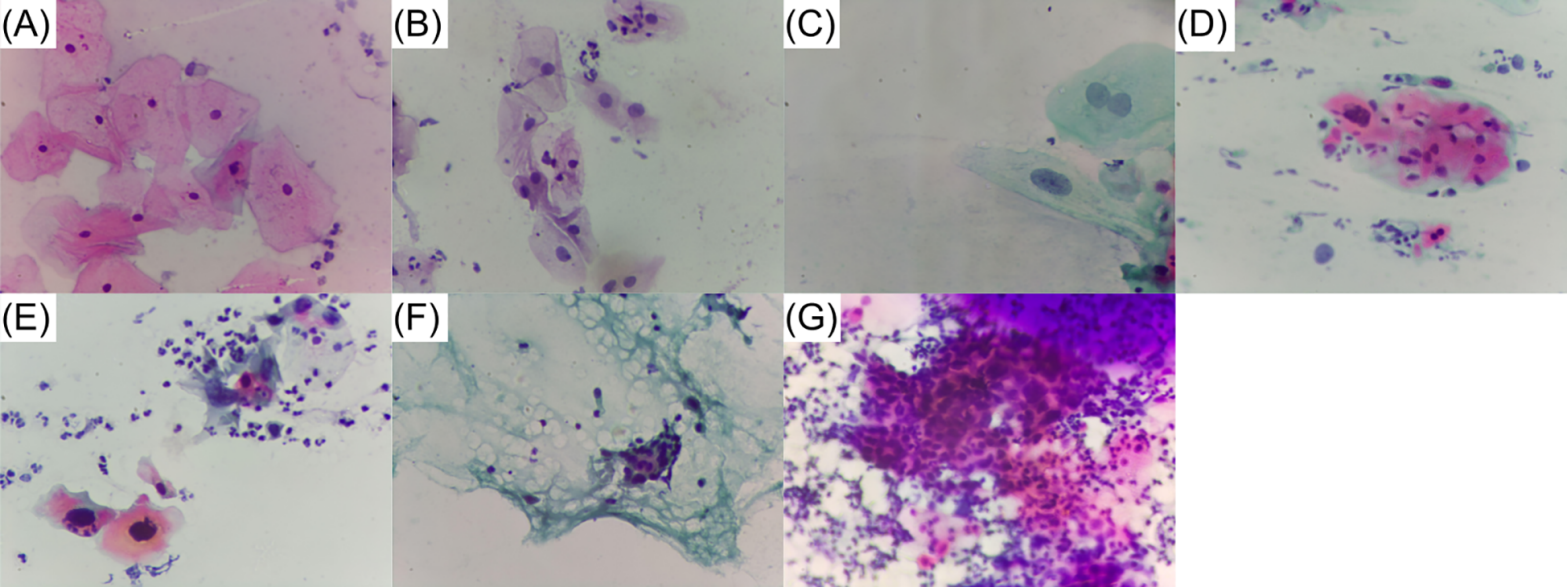
Examples of the different cell types subclasses comprising our custom dataset. **(A)** Normal superficial. **(B)** Normal intermediate. **(C)** Low grade lesion. **(D)** High grade lesion. **(E)** In situ cancer. **(F)** Invasive cancer. **(G)** Adenocarcinoma.

The image dataset used in this study was curated by expert cytotechnologist Guillermo Domínguez under the supervision of pathologist Saúl Tobar, both full time employees at the Hospital General de Zona No. 2 under the Instituto Mexicano del Seguro Social, IMSS (Mexican Institute of Social Security) in Tuxtla Gutiérrez, Chiapas, México. More than 350 cytology slides, borrowed from the hospital and from Dr. Tobar’s private practice, were used. Complete identity privacy was maintained, since we only had access to the cytologic diagnosis of each slide. Cytology slides were manually scanned to find fields of view (FoV) containing cells representative of the most common cell types relevant for CC detection. Digital photos were captured using the microscope described in Section 2.1 and classified as a whole, according to one of the seven subclasses shown in Table 1. These subclasses can be associated with the Bethesda system, the most commonly used framework for reporting cervical cytologic diagnoses (39), as shown in Table 1. Additionally, it is important to mention that each image may comprise multiple cells and various cellular types, however, the definitive classification was based on the presence of the more advanced lesion identified in each image. Examples of the different subclasses are shown in Figure 4. Finally, from the seven subclasses, three major classes were defined: (1) Normal class, (2) Lesion class, and (3) Cancer class. This major classification was proposed in order to maintain a high and balanced number of images per class and taking into consideration the similarities of cell types grouped in each class.

**Table 1:** Correspondence between categories in our custom dataset and the Bethesda system, along with the number of images per class.

Custom dataset	Bethesda system equivalent	# of images
Normal class		655
1. Normal superficial squamous	Negative for intraepithelial lesion or malignancy (for squamous cells)	359
2. Normal intermediate squamous	Negative for intraepithelial lesion or malignancy (for squamous cells)	296
Lesion class		655
3. Low grade lesion	Low-grade squamous intraepithelial lesion	376
4. High grade lesion	High-grade squamous intraepithelial lesion	279
Cancer class		655
5. In Situ cancer	Squamous cell carcinoma	85
6. Invasive cancer	Endocervical adenocarcinoma	276
7. Adenocarcinoma	Endometrial adenocarcinoma	294
		Total: 1965

### 2.4 Model training and evaluation workflow

To implement and evaluate the models described earlier, the dataset is usually divided into training, validation, and testing sets, each set being completely independent from the others. The training set serves as the primary data used to train the CNN model. During training, the model learns to recognize patterns and features by adjusting its weights and biases to minimize the loss function. The validation set is used to monitor the model’s performance during training, enabling hyperparameter tuning and the selection of the best model checkpoint. Finally, the test set is reserved for evaluating the final model’s performance on completely unseen data. Both the validation and testing sets are critical for ensuring the model’s ability to generalize to new, unseen data.

In order to achieve a robust set of hyperparameters, we performed a 5-fold cross-validation for hyperparameter tuning, as follows. The full dataset, previously described, was divided into five groups, each containing three directories corresponding to the three major classes: Normal, Lesion, and Cancer, with 131 images per class. In each fold, images from four of the five groups (80% of the dataset) were combined to form the training set. This training set was then augmented (as described below), resulting in 9,432 training images. The remaining group (20% of the dataset) was further split randomly, with 70% of its images (275 images) assigned to the validation set and the remaining 30% (118 images) to the test set, while maintaining a homogeneous class distribution. Once the training, validation, and test sets were defined, a training and evaluation process was performed on the validation set. This process was repeated five times, each time rotating the group used for validation. Average accuracy values across the five folds were used to determine the best set of hyperparameters. The best-performing model from each fold was saved and later used to evaluate model performance on its corresponding test set, thereby assessing the models’ generalization capability. It is important to note that hyperparameter tuning was performed only on the MobileNet architecture and the resulting hyperparameters were reused for other architectures. This decision was made due to limited computational resources and preliminary tests that identified MobileNet as the best-performing architecture. The reuse of these hyperparameters across other architectures was further justified by their satisfactory performance during validation and testing, as well as the transferability of hyperparameters across similar models trained on the same dataset and task.

**Figure 5:**
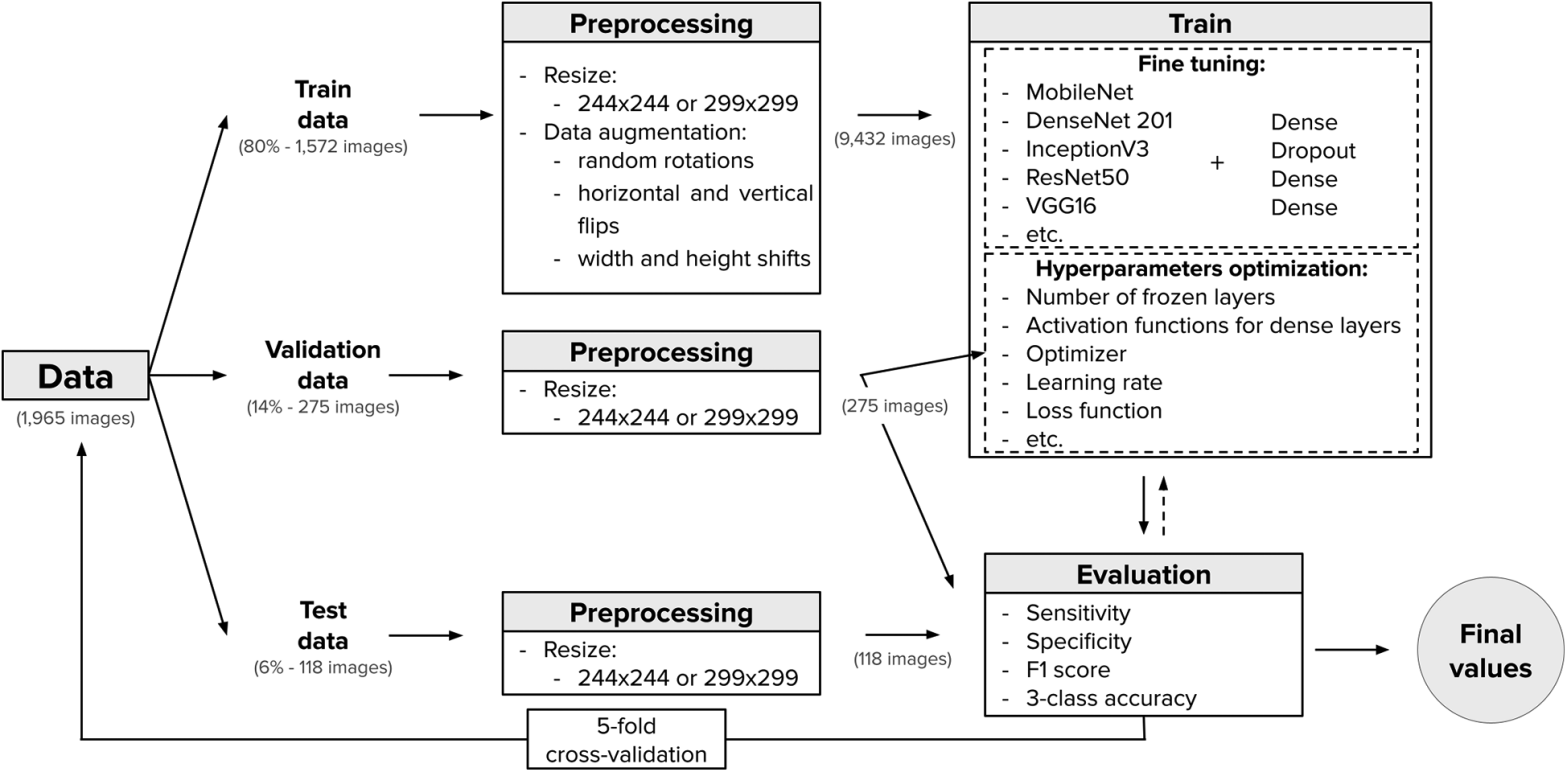
Training-testing workflow.

As shown in Figure 5, it is important to note that the high-quality images captured were resized to either 244×244 or 299×299 pixels to match the input requirements of the different CNN architectures. This reduction in resolution was necessary to ensure compatibility with the pretrained models. After resizing, and as previously mentioned, the training set was augmented to enhance the model’s generalization capabilities. The augmentation techniques applied included random rotations, horizontal and vertical flips, and width and height shifts. Other common augmentation techniques, such as zoom and brightness adjustments, were intentionally excluded to maintain the standardized magnification and illumination conditions achieved by capturing all images with the developed microscope. Importantly, no augmentation was applied to the validation or test sets, as their purpose is to evaluate the model’s performance on realistic, unmodified data. Augmenting these sets would artificially inflate performance metrics, making it difficult to assess the model’s true generalization ability. Although the validation and test sets may appear small, the 5-fold cross-validation approach provides robust results by increasing the number of images evaluated across folds.

The best set of hyperparameters and other tunable considerations were found by iterating over varying configurations of the following: (1) number of trainable layers of the base model (0, 20, 40, 60), (2) activation functions of the classification head (ReLU, LeakyReLU), (3) optimizers (Adam, SGD), (4) learning rate ($10^{-5}$, $10^{-4}$, $10^{-3}$, and $10^{-2}$), (5) batch size (16, 32, 64), (6) number of epochs (10, 20, 50, 100), (7) classification head dimensions (number of layers: 1–3, units per layer: 128, 256, 512 and droput: 0.1, 0.25, 0.4).

The general workflow for training and testing the models is illustrated in Figure 5, and the best-performing hyperparameters, along with the model’s head architecture, are summarized in Table 2. All algorithms were implemented in Python 3.10 using TensorFlow 2.13.1 on a desktop computer equipped with an AMD Ryzen 5 PRO 5,650 G CPU, Radeon graphics, and 16 GB of RAM.

**Table 2:** Best-performing hyperparameters and model head architecture.

Hyperparameter	Optimal value
BM trainable layers	40
Head activation functions	LeakyReLU
Optimizer	Adam
Learning rate	0.001
Batch size	32
Number of epochs	50
Head architecture	Flatten – Dense (512) – Dropout (0.25) – Dense (512) – Dense (128) – Dense (3 – Softmax) – Output (3 Classes)

### 2.5 Exploring CNN classification mechanisms with SVM and Ablation-CAM

Even with their proven efficiency for image classification, CNNs have been criticized for operating as black boxes (48). They receive an input image and output a corresponding class, but the specific characteristics they use for classification are not available. This may sometimes represent a limitation, because it is difficult to know if the algorithm is using valid information for the given context. To further investigate whether our image classification model is focusing on relevant cytological features, we conducted two parallel tests: a Support Vector Machine (SVM) applied to color histograms and the Ablation-CAM technique.

SVM algorithms have been effective in image classification tasks where color is a dominant feature (49). Therefore, in order to inspect the importance of color in classification, we trained and tested an SVM algorithm using color histograms from the dataset, assessing various bin sizes.

On the other hand, ablation-CAM is a technique used to visually recognize which parts of an image are most important for a CNN’s classification decision (50). It generates a heatmap that highlights parts of the images that contributed the most to the network’s prediction. In general terms, Ablation-CAM works by systematically ablating different parts of the feature map in the last convolutional layer and observing how the network’s confidence in its prediction changes. If removing a specific area causes the network to change its prediction significantly, the associated area is likely important for the decision. We applied the Ablation-CAM algorithm to a sample of abnormal images from our dataset, ensuring diversity in coloration and morphology within each class.

## 3 Results

**Figure 6:**
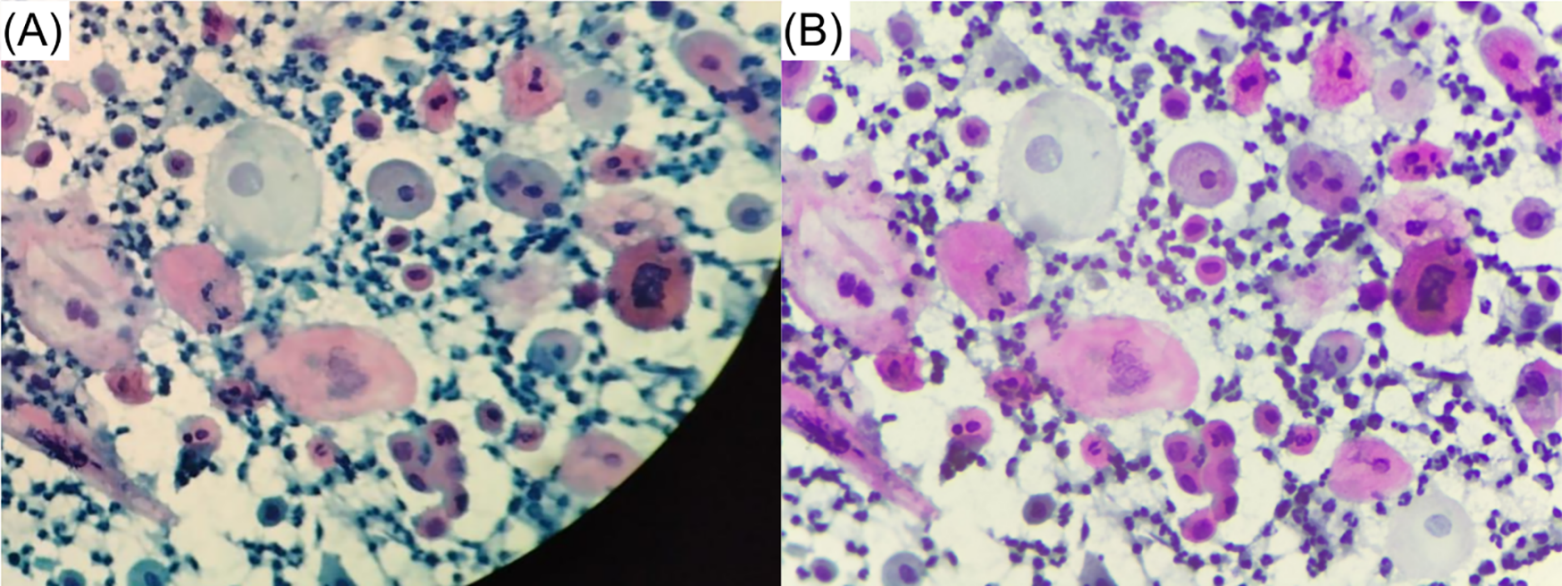
Image comparison of the same FoV captured using: **(A)** an Olympus CX31 and **(B)** our custom-developed microscope.

For assessing the optical capabilities of the developed microscopy platform for cervical cytology analysis, Figure 6, presents a comparison between two images of the same FoV: one captured using a 40X objective of an Olympus CX31 (A) (a widely used brightfield microscope in cytological analysis laboratories within Mexican health institutions), and the other captured using our custom-developed microscope (B). It is important to note that, due to the differences in the optical setup, the FoV of the developed microscope differs from that of common brightfield microscopes. The FoV of the Olympus CX31 with a 40X objective is a circular area of approximately 615 µm in diameter, covering an area of approximately 0.3 ${\mathrm{mm}}^{2}$. In contrast, the FoV of our custom microscope with a 20X objective is limited by the sensor which is placed at the plane where the image is formed by the objective. The sensor captures a rectangular area of $378\times 274\;\mu m^{2}$, covering around 0.1 ${\mathrm{mm}}^{2}$. In fact, the image in Figure 6A was cropped to match the FoV captured by our device, shown in Figure 6B.

An experienced pathologist evaluated both images and exerted the following comments. Firstly, he considered the quality of the image captured with the custom microscope as slightly superior, based on sharpness. Secondly, he commented that he is able to see sufficient details for cytological analysis, identifying two abnormal cells in the image: a koilocyte and an abnormal cell that might be representative of a high-grade lesion.

To supplement the qualitative assessment conducted by the pathologist and obtain a quantitative comparison of the two images, we conducted a BRISQUE (Blind/Referenceless Image Spatial Quality Evaluator) test (51). Although the BRISQUE algorithm is not specifically trained for evaluating cellular images, it extracts features that can be used to assess quality characteristics for arbitrary images. The resulting scores were 26.2 for the custom microscope and 46.7 for the Olympus microscope. Since lower BRISQUE scores indicate better image quality, these results further support the conclusion that the images obtained with our custom microscope are of acceptable quality compared to those captured using a commercial microscope used for cytologic analysis.

To evaluate the performance of our pre-trained models listed above, we have chosen four metrics: sensitivity (Sens), specificity (Spec), $F_{1}$-score (F1), and accuracy (Acc). Sensitivity tells us the proportion of actual positive cases (abnormal cells) correctly identified by the test. This is a crucial criterion since, in the context of CC screening, the consequences of a false negative can have substantial critical health risks and economic burdens. Specificity represents the proportion of actual negative cases (normal cells) correctly identified by the test. Specificity, although not as critical as sensitivity, is also a relevant criterion since it can trigger unnecessary stress to the patient and give additional work to the specialist. $F_{1}$-score is a measure of predictive performance often used in medical image analysis research. Finally, the accuracy represents the overall proportion of correct results. Given the rationale of our proposal, which will be further discussed in the following section, some of the computed metrics allow for confusion between the two considered abnormal classes.

(1) $$\begin{array}{cc} \mathrm{Sensitivity} & =\frac{{\mathrm{TP}}_{\mathrm{les}}^{\prime }+{\mathrm{TP}}_{\mathrm{can}}^{\prime }}{{\mathrm{Tot}}_{\mathrm{les}}+{\mathrm{Tot}}_{\mathrm{can}}}\\ \mathrm{Specificity} & =\frac{\mathrm{TN}}{{\mathrm{Tot}}_{\mathrm{nor}}}\\ F_{1} & =\frac{2({\mathrm{TP}}_{\mathrm{les}}^{\prime }+{\mathrm{TP}}_{\mathrm{can}}^{\prime })}{2({\mathrm{TP}}_{\mathrm{les}}^{\prime }+{\mathrm{TP}}_{\mathrm{can}}^{\prime })+{\mathrm{FP}}_{\mathrm{les}}^{\prime }+{\mathrm{FP}}_{\mathrm{can}}^{\prime }+\mathrm{FN}}\\ \mathrm{Accuracy} & =\frac{{\mathrm{TP}}_{\mathrm{nor}}+{\mathrm{TP}}_{\mathrm{les}}+{\mathrm{TP}}_{\mathrm{can}}}{{\mathrm{Tot}}_{\mathrm{nor}}+{\mathrm{Tot}}_{\mathrm{les}}+{\mathrm{Tot}}_{\mathrm{can}}} \end{array}$$

In Equation 1, we can see the mathematical definitions of the used evaluation metrics. ${\mathrm{TP}}_{\mathrm{les}}^{\prime }$ and ${\mathrm{TP}}_{\mathrm{can}}^{\prime }$ are true positives of the lesion and cancer classes respectively, allowing for confusion with the other positive class. $F_{1}$-score is computed under the same flexibility. On the other hand, specificity and accuracy are defined in the conventional way. Additionally, a 5-fold cross-validation was performed in all tests in order to assess generalization performance. Therefore, all reported values correspond to the average across the five different runs.

The results for the chosen evaluation metrics are presented in Table 3 for the three best-performing models: MobileNet, DenseNet201 and InceptionV3. All three models exhibited consistently high sensitivity values. MobileNet achieved the highest scores, with 98.26% sensitivity on the validation set and 97.95% on the test set. DenseNet201 followed with 96.19% and 96.67% for the validation and test sets, respectively, while InceptionV3 showed sensitivity values of 95.87% on the validation set and 93.08% on the test set. These results suggest that MobileNet may be more robust in detecting subtle abnormalities in cervical cell images.

**Table 3:** Evaluation metrics for the top-3 evaluated models for validation and test sets.

Base model	Set	Sens	Spec	$F_{1}$	Acc
		(%)	(%)	(%)	(%)
MobileNet	V	98.26	88.91	96.42	87.32
	T	97.95	88.72	96.23	86.15
DenseNet201	V	96.19	91.96	96.09	88.98
	T	96.67	90.26	95.93	85.82
InceptionV3	V	95.87	91.30	95.76	87.02
	T	93.08	91.79	94.29	84.95

From Table 3, we can also notice that, contrary to the high sensitivity values, specificity was relatively lower for all models. This indicates a higher likelihood of false positives, meaning normal cells were more often misclassified as abnormal. Among the models, MobileNet performed the worst in terms of specificity, while InceptionV3 showed the best performance. The $F_{1}$-score for all three models were consistently high, indicating a good balance between precision and sensitivity. Notably, MobileNet achieved a slightly higher $F_{1}$score, further reinforcing its effectiveness in identifying abnormal cells. Finally, we can notice that accuracy values are considerably lower which, in conjunction with the sensitivity values, can be explained as misclassifications between the two abnormal classes.

Table 4 displays the precision values for each cellular type across the three models. Notably, MobileNet achieves excellent identification for all cancer subcategories and high-grade lesions, which are the most critical abnormal classes. However, its performance on the low grade lesion and normal subclasses is relatively weaker. In contrast, DenseNet201 presents optimal performance only on adenocarcinoma subclass, while InceptionV3 presents excellent performance only on adenocarcinoma and in situ classes.

**Table 4:** Breakdown of precision for identification with MobileNet base model for each cellular type contained in the custom dataset.

C.T.	Set	MobileN	DenseN	Inception
		(%)	(%)	(%)
Sup	V	90.44	93.99	92.25
	T	92.88	92.38	92.65
Int	V	87.33	89.31	89.94
	T	83.78	87.98	91.91
L.G.	V	95.96	93.63	92.07
	T	93.04	93.11	88.87
H.G.	V	98.87	95.66	97.15
	T	100.00	97.32	91.77
In Situ	V	98.00	96.53	98.18
	T	100.00	89.33	100.0
Inv	V	99.00	96.61	95.27
	T	100.00	98.95	93.45
Adeno	V	100.00	100.00	99.0
	T	100.00	100.00	99.05

**Figure 7:**
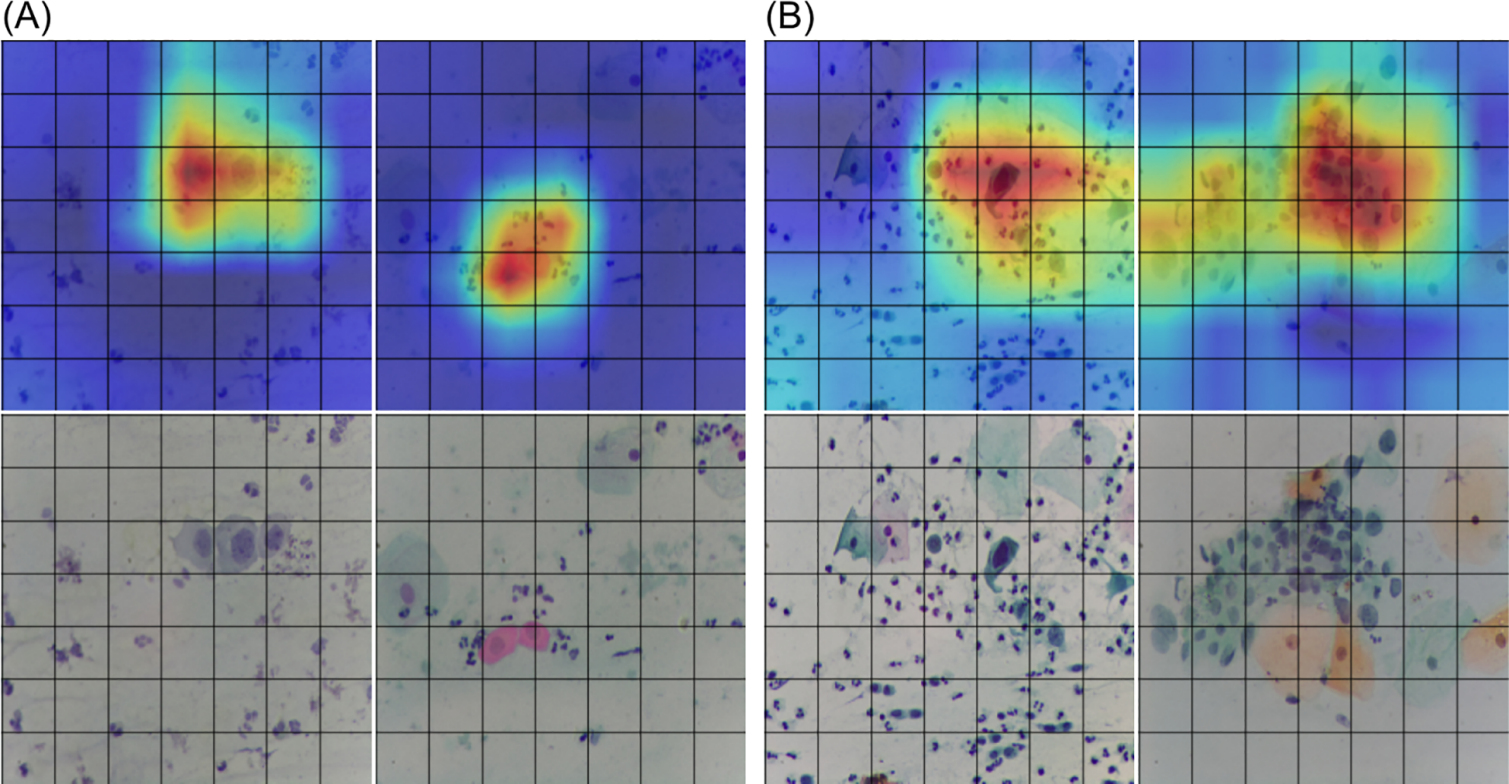
Comparison of Ablation-CAM results alongside their corresponding original images for: **(A)** low grade lesions, and **(B)** cancerous stage.

Regarding the SVM test for understanding the classifying mechanisms of CNNs, the best performance was achieved with 50 bins per color band. Yielding a sensitivity of 84.78%–84.62%, specificity of 76.52%–73.33%, and an overall accuracy of 67.25%–65.47% for the validation and test sets, respectively. While these results were significant, they were notably lower than those of the CNN models, indicating that while color contributes to classification, the CNNs are recognizing more complex, higher-order features beyond color alone. As for the Ablation-CAM results, in Figure 7, we present a comparison of the Ablation-CAM outcomes alongside their corresponding original images. The “hot” areas on the heatmaps indicate the regions of the images that are most relevant for the achieved classification. The first two, Figure 7A are images classified by the specialists as low grade lesions, while the last two, Figure 7B, were identified as cancerous. We can observe that the heatmaps effectively highlight regions containing abnormal cells while avoiding empty regions or those with normal cells. These qualitative results support the conclusion that MobileNet is extracting meaningful information from cellular patterns.

**Figure 8:**
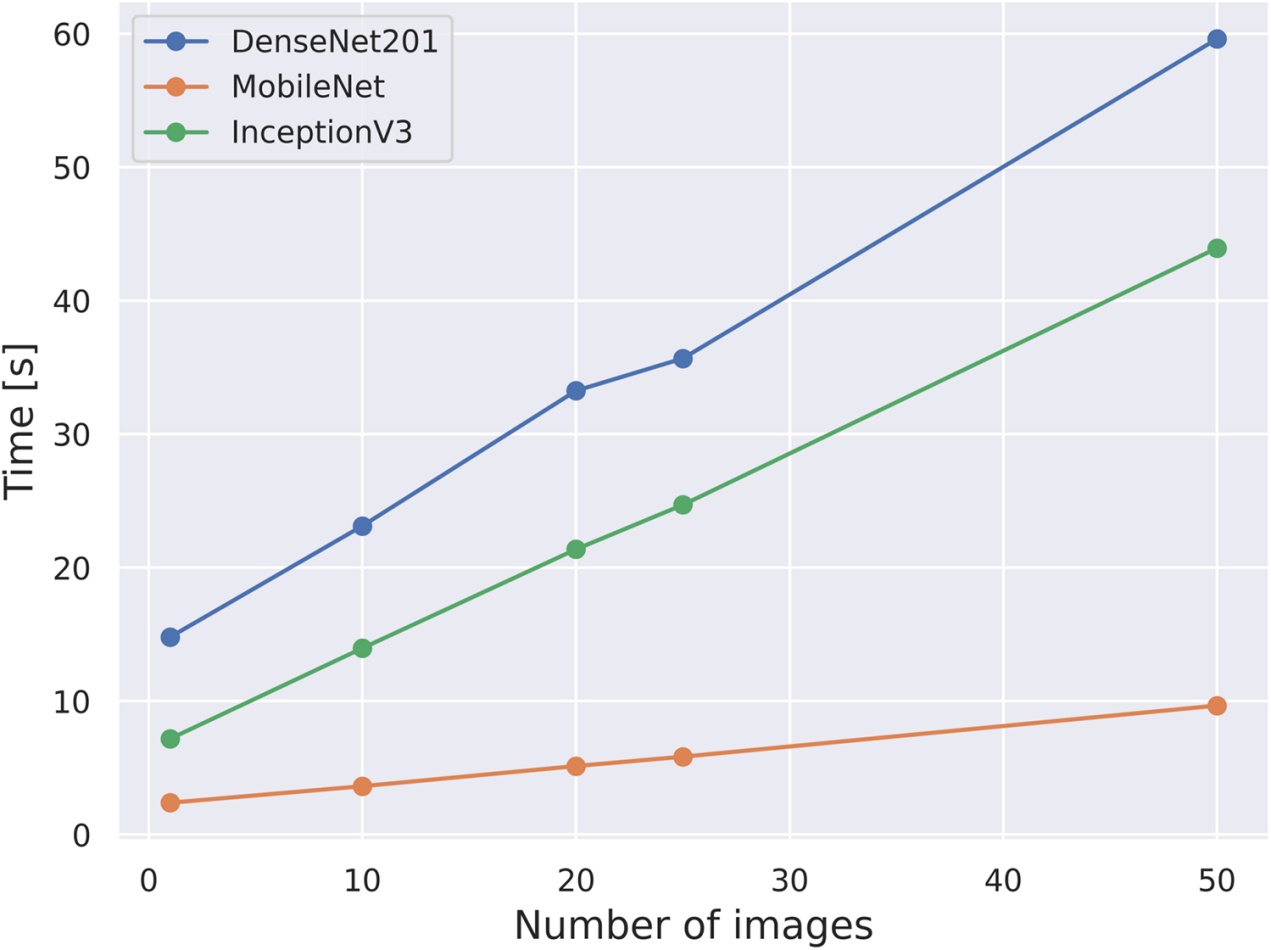
Computation times in seconds on a Raspberry Pi4 8 Gb for the top-3 models for classifying different number of images.

In addition to classification performance, another important aspect to consider when deploying a device like the one proposed in this paper, is computing time. As mentioned earlier, the core of the platform’s software and control is a Raspberry Pi 4, which, while offering some computing power, may not be sufficient for real-time AI applications. To evaluate the computational efficiency, we conducted timing tests for the models that performed the best on the classification task. Different batches of 1, 10, 20, 25, and 50 images were classified by the trained models in order to check classification times as a function of the number of images running on a Raspberry Pi. Results shown in Figure 8, also point to MobileNet as the best-performing model based on this criterion. It runs considerably faster, up to six times faster than DenseNet201, which was ranked as the second best model. MobileNet took about 10 s to classify 50 FoV images, which translates to approximately 200 ms per FoV image.

## 4 Discussion

The contribution of this research can be divided into two main aspects: (1) the development of an automated, low-cost microscopy device, and (2) its application in creating an algorithm to identify abnormal cell types relevant to cervical cytological analysis.

Regarding the first aspect, the previously described device was designed to perform automated scanning of Pap smears in resource-constrained settings. In contrast to other published automated microscopes, our prototype supports automated scanning of a $50\times 25\;{\mathrm{mm}}^{2}$ surface, which is required for conventional Pap smears reading. It is constructed using 3D-printed parts and simple mechanical components, which allows to maintain a production cost below 500 USD, making it a cost-effective proposal. Additionally, it incorporates a software that is designed for intuitive use by non-specialists. These characteristics highlight the described platform as a valuable asset for reading Pap smears in low-resource settings.

In the second aspect, we used our microscopy device for developing a CNN-based classification system that identifies the most important abnormal cell types relevant to a cervical cytological analysis. The results presented in the previous section identify MobileNet as the best-performing model for detecting the five kind of abnormal cells considered in this work. Compared to other models, it shows the highest sensitivity and F1 scores. Table 3 details how well each model classifies individual subclasses. From this data, we can conclude that the main area for improvement with MobileNet lies in distinguishing between normal cells and low-grade lesions. This challenge is understandable due to the subtle and often variable morphological changes associated with low-grade lesions. Expanding the training dataset with more examples of these cases is expected to improve classification accuracy. Furthermore, the SVM and Ablation-CAM tests results suggest that MobileNet architecture is accurately using cellular features for performing the classification of full FoV images. This is an important validation component, that to our knowledge, had not been performed on classification researches that work with full FoVs. However, since a full dataset-wide analysis was beyond the scope of this study, we did not apply Ablation-CAM to all images in our dataset and further investigation in this regard is left for future work.

On the other hand, it is important to acknowledge the relatively low specificity achieved by the tested models, particularly MobileNet, which exhibited the highest sensitivity values. A specificity of 90% implies that approximately 10% of fields containing only normal cells would be misclassified as abnormal. Given that each slide consists of approximately 12,500 fields of view (FoVs), a hypothetical normal sample would result in several hundreds of mistakenly flagged images. This would require manual review of all these fields, effectively defeating the purpose of the device. To mitigate these challenges, there are a number of possibilities that should be explored. First, enriching the training dataset with more images is essential. It has been extensively documented that CNNs improve their accuracy when trained on sufficiently large and diverse datasets (52, 53). In this study, we curated a relatively modest dataset of 1,600 images, which is small considering the complexity of cytological samples. Second, an alternative approach would be to develop an asymmetrical classifier that treats false positives and false negatives differently (14). While allowing a model to misclassify a substantial fraction of malignant cells as normal may seem counterintuitive, it could still be effective. Theoretically, if we have dozens of abnormal cells in an abnormal slide, which is typically the case, even with a high false negative rate, the system would detect a percentage of malignant cells, allowing the sample to be properly classified as positive. Similarly, another approach is to count the number of detected abnormal cells and classify the specimen as suspicious if it exceeds a certain threshold. Finally, a commonly proposed solution is to integrate the system with parallel human screening. However, this increases workload and costs, undermining the system’s intended purpose. It is worth noting that ThinPrep, one of the commercially available system, is primarily marketed for use in this manner. To conclude, we would like to state that regardless of the best approach to address the challenge of low specificity, an important idea behind our proposal is that images acquired in rural or underserved areas—where trained technicians and specialists for Pap smear analysis may be unavailable—could be uploaded to the cloud. This would enable remote specialists to review digitized images of suspicious FoVs, facilitating assessment without the need to transport personnel or physical samples.

Next, to contextualize the results obtained in this research within the existing literature, it is important to note that, unlike most classification studies that focus on single-cell images, our results are based on full FoV images, which may contain several cells. Therefore, direct comparison with other studies is not always straightforward. However, we can compare our work with a few studies that take a similar approach. Hussain et al. (43) reported a four-class classification of full FoV images, achieving an accuracy of 98.9% and a sensitivity of 97.8%, using a combination of proprietary and public datasets. Similarly, Alsalatie et al. (44) worked with full FoV images from liquid-based cytology slides, achieving an accuracy of 99.6% for a four-class classification. In contrast, our study integrates more classes, including three cancer categories, two lesion classes, and two normal classes, which we group into three major classes to balance the dataset and group cell types with similar characteristics. Furthermore, our dataset consists on pictures taken from conventional Pap smears, which is the more common technique used in LMICs. For real-life applications, distinguishing between normal and abnormal cell types is more critical than achieving extreme precision across multiple subcategories. It is also worth mentioning that we also conducted a two-class classification, but the results were slightly inferior to the three-class approach. This could be due to the unbalanced number of images between the two classes or the grouping of cell types with less similar characteristics. In this context, it is important to highlight that most recent researches in the field focus on developing sophisticated algorithms for tasks such as object detection, segmentation, image classification, or combinations of these (25, 54). For example, Zhu et al. (55) developed a system for automated cervical cytology screening that integrates five complex AI models and classifies samples according to The Bethesda System. While this system achieved impressive results, it requires significant computing resources, which may not be feasible in low-resource settings.

While our study uses simpler classification algorithms, one of the core strengths in this proposal is to compensate for algorithmic complexity with the development of a robust, standardized dataset. There is evidence that changes in some of these factors might affect performance (56, 57). In this context, it is relevant to highlight some noteworthy advantages of our custom dataset when compared with other publicly available datasets. Firstly, all images were captured using our custom microscope which is translated into standardized magnification, aspect ratio, illumination conditions, and camera settings. Secondly, the inclusion of a diverse set of more than 350 conventional cytology slides, borrowed from IMSS, and from Dr. Tobar private practice, enables us to account for variations in sample preparations. Additionally, although staining technique is standardized to the conventional Papanicolaou protocol, slight variations are present due to differences in chemical brands and personal preferences. These considerations make our database a robust foundation for generalization and model performance. Additionally, the utilization of a Raspberry HQ camera allowed it to have high resolution images of 4056×3040 pixels. This standardized dataset and meticulous collection methodology lay a solid groundwork for effective image classification within our study.

Despite the benefits of this dataset and the use of simpler classification algorithms, there remain challenges for real-life implementation. One critical consideration is the time required to process a slide. Given that Papanicolaou slides cover an area of approximately $50\times 25\;{\mathrm{mm}}^{2}$, and considering that the platform’s FoV is $378\times 274\;\mu m^{2}$, the device would need to capture and classify roughly 12,500 images to fully scan a slide. The key factors determining time estimation include travel between FoVs, image capture at each FoV, and all steps involved in image classification. Based on the characteristics of our electromechanical setup, the full slide could theoretically be scanned in under five minutes. On the other hand, considering the exposure times required for optimal image quality with our illumination setup, capturing all images should take no more than 15 s. However, in practice, we have not achieved scanning speeds below 60 min, primarily due to the SD card’s writing speed. This limitation could be approached by using a faster SD card or capturing lower-resolution images, especially since we already downsample images for classification. An additional factor that significantly impacts processing time and viability, yet has not been addressed in this work, is the need for autofocus. As the system moves across different FoVs, variations in the focal plane are inevitable. This underscores the importance of implementing an autofocus function to maintain image clarity. While we have not yet incorporated autofocus, the microscope’s mechanical and electronic design fully supports its integration in future iterations. Furthermore, using MobileNet for the classification task alone would take approximately 45 min, not accounting for the additional time required for image capture and resizing. Taking these additional steps into account, the total processing time could increase significantly, potentially limiting the device’s practicality in real-world settings. To address this limitation, increasing the system’s computing power appears to be a promising solution. Options such as a Coral accelerator or a Raspberry Pi AI kit module, each costing around 70 USD, could considerably reduce the classification time. With this increased computing power, it might also be feasible to implement more sophisticated computer vision algorithms, thereby improving both the precision and efficiency of the classifier.

Another important consideration for real-life use is the huge diversity of elements that appear when reading a full slide. While our algorithm has been trained to recognize seven specific cellular types, the device will inevitably encounter additional scenarios it has not been trained for, such as images containing no cells, overly saturated images with no visibility, or fields containing blood, mucus, immune cells, or other types of artifacts. Additionally, including normal endocervical and metaplastic cells is crucial, as their presence is a requirement for slide adequacy according to the Bethesda system. However, we believe that being an open-source, reproducible device could foster the development of a collaborative, standardized dataset that includes all potential cellular types encountered in cervical cytological analysis. This would not only improve the model’s accuracy but also enhance its applicability in diverse clinical environments.

While our study shows promising results towards developing an integrated automated system for cervical cytologic analysis, real-world implementation requires solving several additional challenges. At the same time, the cost of achieving full coverage of cervical cytology screening under the traditional framework remains prohibitive in LMICs. As highlighted earlier, countries with significant rural populations or complex geographies find that the traditional screening process is far from cost-effective. The low-cost, automated microscopy device we propose, combined with deep learning algorithms, offers a potential solution to these obstacles. While this prototype is only a first step, it demonstrates how new technologies can begin to alleviate the burden on resource-constrained settings, extending access to vital screening services and ultimately improving health outcomes for underserved populations.

## 5 Conclusion

In this study, we developed a low-cost microscopy platform equipped with AI-based computer vision capabilities for cervical cytology screening. The microscopy platform, costing below 500 USD per unit, has sufficient optical capabilities for analyzing cervical cells in Pap smears and it is capable of scanning a chosen area inside a $50\times 25\;{\mathrm{mm}}^{2}$ area. The platform was used for building a dataset of 1,965 images spanning seven different cellular types relevant in cervical cytology analysis. Furthermore, this dataset was used to train and test several CNN algorithms for automated classification of the different seven cellular types within three major classes. Through the comparative evaluation of various models, MobileNet emerged as the most effective choice, achieving high sensitivity and competitive F1 scores, which are critical for minimizing false negatives. Additionally, timing tests demonstrated that MobileNet significantly outperformed other models in computational speed, making it a viable candidate for real-time applications, particularly when paired with computing accelerators like the Coral and Raspberry Pi AI modules.

In conclusion, our platform presents a promising step toward automating cervical cytology analysis in low-resource settings, where traditional methods may be limited. By optimizing both the hardware and the AI models, it has the potential to significantly reduce the time, costs and effort required for initial screening, while ensuring accurate detection of abnormal cells. Future work will focus on refining the system for full-slide analysis, increasing model robustness, and performing field testing to ensure that the platform meets the demands of real-world clinical applications in real settings.

## Data Availability

The raw data supporting the conclusions of this article will be made available by the authors, upon reasonable request.
